# Coactosin Phosphorylation Controls Entamoeba histolytica Cell Membrane Protrusions and Cell Motility

**DOI:** 10.1128/mBio.00660-20

**Published:** 2020-08-04

**Authors:** Muhammad M. Hasan, José E. Teixeira, Ying-Wai Lam, Christopher D. Huston

**Affiliations:** a Department of Medicine, University of Vermont, Larner College of Medicine, Burlington, Vermont, USA; b Cellular, Molecular, and Biomedical Sciences Graduate Program, University of Vermont, Burlington, Vermont, USA; c Proteomics Facility, Vermont Genetics Network, University of Vermont, Burlington, Vermont, USA; d Department of Biology, University of Vermont, Burlington, Vermont, USA; University of California, Los Angeles

**Keywords:** ADF/cofilin, coactosin, *Entamoeba histolytica*, amoebiasis, cell motility, phosphoproteomics

## Abstract

Invasion of the colon wall by Entamoeba histolytica during amoebic dysentery entails migration of trophozoites through tissue layers that are rich in extracellular matrix. Transcriptional silencing of the E. histolytica surface metalloprotease EhMSP-1 produces hyperadherent less-motile trophozoites that are deficient in forming invadosomes. Reversible protein phosphorylation is often implicated in regulation of cell motility and invadosome formation. To identify such intermediaries of the *EhMSP-1*-silenced phenotype, here we compared the phosphoproteomes of *EhMSP-1*-silenced and vector control trophozoites by using quantitative tandem mass spectrometry-based proteomics. Six proteins were found to be differentially phosphorylated in *EhMSP-1*-silenced and control cells, including EhCoactosin, a member of the ADF/cofilin family of actin-binding proteins, which was more frequently phosphorylated at serine 147. Regulated overexpression of wild-type, phosphomimetic, and nonphosphorylatable EhCoactosin variants was used to test if phosphorylation functions in control of E. histolytica actin dynamics. Each of the overexpressed proteins colocalized with F-actin during E. histolytica phagocytosis. Nonetheless, trophozoites overexpressing an EhCoactosin phosphomimetic mutant formed more and poorly coordinated cell membrane protrusions compared to those in control or cells expressing a nonphosphorylatable mutant, while trophozoites overexpressing nonphosphorylatable EhCoactosin were significantly more motile within a model of mammalian extracellular matrix. Therefore, although EhCoactosin’s actin-binding ability appeared unaffected by phosphorylation, EhCoactosin phosphorylation helps to regulate amoebic motility. These data help to understand the mechanisms underlying altered adherence and motility in *EhMSP-1*-silenced trophozoites and lay the groundwork for identifying kinases and phosphatases critical for control of amoebic invasiveness.

## Abstract (precis)

Invasive amoebiasis, caused by the intestinal parasite Entamoeba histolytica, causes life-threatening diarrhea and liver abscesses, but, for unknown reasons, only approximately 10% of E. histolytica infections become symptomatic. A key requirement of invasion is the ability of the parasite to migrate through tissue layers. Here, we systematically looked for differences in protein phosphorylation between control parasites and a previously identified hyperadherent E. histolytica cell line that has reduced motility. We identified EhCoactosin, an actin-binding protein not previously known to be phosphoregulated, as one of the differentially phosphorylated proteins in E. histolytica and demonstrated that EhCoactosin phosphorylation functions in control of cell membrane dynamics and amoebic motility. This and the additional differentially phosphorylated proteins reported lay the groundwork for identifying kinases and phosphatases that regulate tissue invasiveness.

## INTRODUCTION

Amoebiasis, due to the protozoan parasite Entamoeba histolytica, is an important cause of life-threatening diarrhea in children under 5 years of age in developing countries. Motile E. histolytica trophozoites colonize the large intestine and, in approximately 10% of infections, invade the colonic mucosa to cause amoebic colitis, characterized by fever, abdominal pain, tenesmus, and dysentery. Occasionally, hematogenous spread results in organ abscesses, such as spread via the portal vein to the liver (1–3). Understanding how and why E. histolytica invades through the colonic epithelium could help explain the variable outcomes of infection.

Studies of the pathogenesis of amoebic colitis suggest a stepwise model of virulence (4). After ingestion of fecally contaminated food or water, excystation of cysts yields motile trophozoites that colonize by adhering to colonic mucus and epithelial cells. Adherence is largely mediated by an *N*-acetyl-d-galactosamine (GalNAc)-specific amoebic surface lectin (5). Host cell killing follows from a variety of mechanisms, including secretion of cytolytic peptides (i.e., amoebapores), a number of secreted and cell surface cysteine proteases, trogocytosis (literally, cell nibbling), and contact-dependent induction of host cell apoptosis (6–9). Phagocytosis of host cells, traditionally considered a defining pathological feature of amoebiasis, appears to occur via two mechanisms, a relatively slow process of trogocytosis whereby pieces of cells are torn off and ingested and a rapid process in which cell-surface alterations on previously damaged (e.g., apoptotic) cells trigger the engulfment of whole cells (9, 10). Not surprisingly, the clinical illness results from a combination of tissue destruction and acute inflammation.

The ability of E. histolytica to invade by migrating through extracellular matrix (ECM)-rich tissues is a requirement for virulence that remains poorly understood. We previously demonstrated that amoebic tissue invasion involves invadosomes, which are specialized F-actin-rich structures that couple ECM degradation with actin-based protrusions at the cell membrane (11). Invadosomes are a conserved feature of tissue-invasive cells. For example, cancer cell invadosomes function during metastasis as sites of coordinated activity of ECM-degrading matrix metalloproteases and adhesive integrins (12, 13). Silencing the expression of an E. histolytica metallosurface protease, EhMSP-1, results in the formation of fewer invadosomes than in control cells; furthermore, it increases amoebic adherence while reducing motility (11, 14). Compensatory changes in cells with constitutively silenced *EhMSP-1* are likely, so it is unclear if EhMSP-1 functions directly in amoebic adherence, motility, and invadosome formation. Regardless, signaling differences in *EhMSP-1*-silenced and control amoebae should inform how E. histolytica adherence, motility, and tissue invasiveness are controlled.

Dynamic cellular processes are often regulated by posttranslational protein modifications, and specifically, reversible protein phosphorylation plays an important role in invadosome formation and regulation in mammalian cells (15). In this study, we identified differentially phosphorylated proteins in *EhMSP-1*-silenced and control trophozoites through a combination of affinity enrichment and mass spectrometry. Six differentially phosphorylated peptides were identified, including peptides from unconventional myosin IB, a Rho guanine nucleotide exchange factor, and the carboxy-terminal domain of EhCoactosin. We chose to focus follow-up experiments on EhCoactosin. Coactosin is not known to be phosphoregulated, but other members of the actin-depolymerizing factor (ADF)/cofilin family are phosphoregulated and function in the control of actin dynamics during cell migration (16–18). Furthermore, EhCoactosin was previously implicated in control of E. histolytica virulence phenotypes (19, 20). Follow-up experiments using trophozoites overexpressing phosphomimetic and nonphosphorylatable EhCoactosin variants confirmed phosphoregulation of EhCoactosin and its involvement in the control of amoebic membrane protrusions (e.g., pseudopods) and motility within an ECM-rich environment. This study, therefore, provides a validated set of E. histolytica phosphopeptides, demonstrates involvement of EhCoactosin phosphoregulation in the control of actin dynamics, and lays the groundwork for identifying kinases and phosphatases critical for control of amoebic virulence.

## RESULTS

### Several proteins, including EhCoactosin, are differentially phosphorylated in *EhMSP-1*-silenced trophozoites.

**FIG 1:**
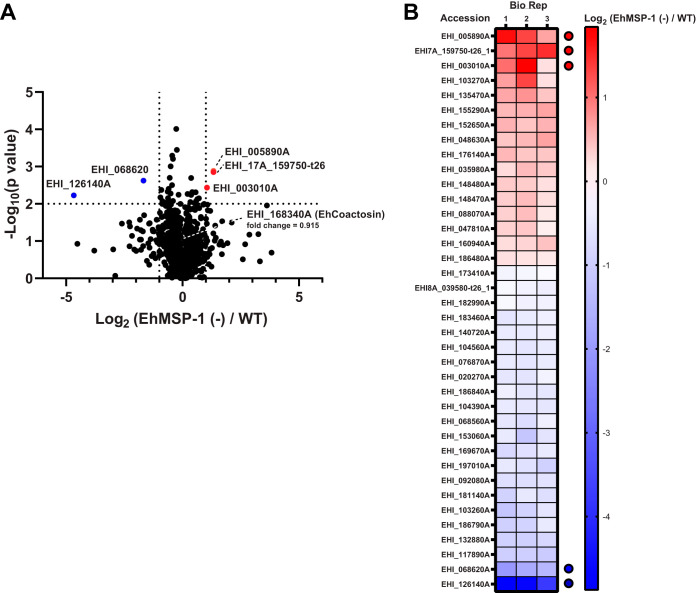
Differential proteome of *EhMSP-1*-silenced E. histolytica. (A) Volcano plot; 892 proteins were identified and quantified by stable isotope dimethyl labeling quantitative proteomics in all three biological replicates comparing *EhMSP-1-*silenced [EhMSP-1 (−)] trophozoites to vector control trophozoites, and 38 proteins were differentially expressed (*P* < 0.01). Five proteins had a >2-fold difference (3 upregulated and 2 downregulated, highlighted in red and blue, respectively). Cutoffs of EhMSP-1 (−)/WT at 2-fold (log_2_ 2 = 1) and *P* value at 0.01 (−log_10_ 0.01 = 2) are indicated by dotted line(s) on the *x* and *y* axes, respectively. See also Table S1 in the supplemental material. (B) Heat map. The log_2_ ratios [EhMSP-1 (−)/WT] of the differentially abundant proteins with a *P* value of <0.01 are represented in a heat map, listed from high to low according to their average log_2_ fold change. The five proteins with a >2-fold difference and a *P* value of less than <0.01 are indicated with red (upregulated) and blue (downregulated) dots, respectively. EhCoactosin (EHI_168340A) was relatively unchanged between WT (light [L]) and *EhMSP-1*-silenced (heavy [H]) parasites (mean H/L = 0.915; *P* = 0.077).

We compared protein expression in *EhMSP-1*-silenced and vector control G3 strain trophozoites using isotopomeric dimethyl labeling (21) followed by quantitative liquid chromatography-tandem mass spectrometry. Eight hundred ninety-two proteins were identified in all three biological replicates, and among these, 38 proteins were differentially abundant (*P* value < 0.01; 16 upregulated and 22 downregulated) (Fig. 1A and B). Five proteins differed greater than 2-fold (3 upregulated and 2 downregulated) (Fig. 1A and B; see also Table S1 and Data Set S1 in the supplemental material) (all mass spectrometry/proteomics data are available via ProteomeXchange with identifier PXD018276).

10.1128/mBio.00660-20.5 TABLE S1 Proteins that are differentially expressed following *EhMSP-1* silencing. Download Table S1, PDF file, 0.1 MB. Copyright © 2020 Hasan et al. 2020 Hasan et al. This content is distributed under the terms of the Creative Commons Attribution 4.0 International license.

10.1128/mBio.00660-20.9 DATA SET S1 Summarized mass spectrometry data. (Sheet one) Proteins detected by mass spectrometry and relative abundance in *EhMSP-1-*silenced and control trophozoites. Proteins with altered abundance are listed in Table S1. (Sheet two) Phosphopeptides detected. Phosphopeptides with a fold change either >1.5-fold or <0.67-fold (CV < 30%) following *EhMSP-1* silencing are listed in Table S3. Mass spectrometry proteomics data have also been deposited to the ProteomeXchange Consortium via the PRIDE partner repository with the dataset identifier PXD018276. Download Data Set S1, XLSX file, 0.3 MB. Copyright © 2020 Hasan et al. 2020 Hasan et al. This content is distributed under the terms of the Creative Commons Attribution 4.0 International license.

**FIG 2:**
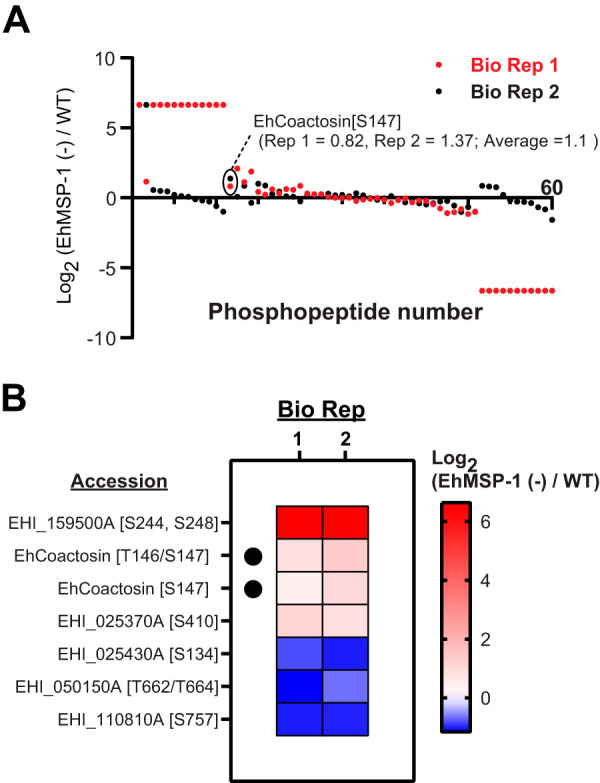
Differential phosphoproteomes of *EhMSP-1*-silenced and control E. histolytica trophozoites. (A) Identification and quantification of phosphopeptides. After phosphopeptide enrichment (IMAC and TiO_2_), 60 dimethyl-labeled phosphopeptides were identified and quantified in both independent biological replicates (Bio Rep 1 and 2; indicated in red and black, respectively). The points indicating log_2_ ratios [EhMSP-1 (−)/WT] corresponding to the EhCoactosin (EHI_168340A) S147-containing phosphopeptide of interest is circled for each biological replicate. (B) Heat map showing the log_2_ ratios [EhMSP-1 (−)/WT] for 6 unique phosphopeptides with a change of either >1.5-fold or <0.67-fold and coefficient of variation of <30% in both biological replicates, listed from high to low according to their average log_2_ fold change. Note that the EhCoactosin S147-containing phosphopeptide (black dots; EHI_168340A) was detected as both a fully trypsin-digested peptide and a miscleaved variant. See also Table S2 in the supplemental material.

**FIG 3:**
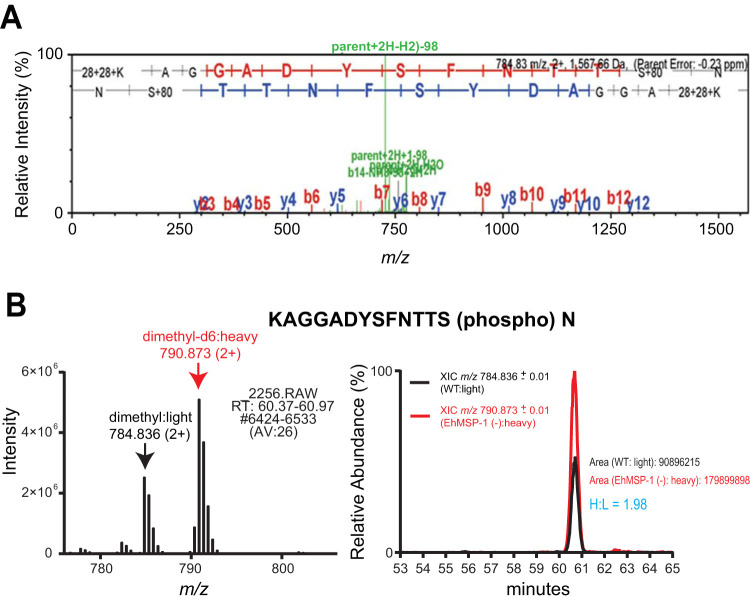
Phosphorylation of EhCoactosin at S147. (A) Identification of EhCoactosin S147-containing phosphopeptide. The coactosin S147 phosphopeptide (EHI_168340A) KAGGADYSFNTTS(phospho)N was identified via SEQUEST with XCorr = 3.31, and precursor ΔM (experimental *m/z* and theoretical *m/z*) = −0.23 ppm. The MS/MS spectrum is annotated by Scaffold. The primary amines at the N terminus and on the lysine residue are dimethyl labeled. (B) Increased abundance of EhCoactosin S147-containing phosphopeptide in *EhMSP-1*-silenced Entamoeba histolytica. The MS1 spectrum of the light- (dimethyl-d0) and heavy-labeled (dimethyl-d6) EhCoactosin S147-containing phosphopeptides and the extracted ion chromatograms showing the light and heavy isotopologues eluting symmetrically at the same retention time are shown on the left and right, respectively. Heavy/light (H/L) was quantified by precursor ion elution profiles to be an average of 1.93 from multiple measurements from both biological replicates (one representative chromatograph [H/L = 1.98] is shown).

We next used a combination of TiO_2_ bead precipitation (22) and Fe-nitrilotriacetic acid (NTA) immobilized metal-ion chromatography (IMAC) columns (23) to selectively enrich phosphopeptides from *EhMSP-1-*silenced and control trophozoites. Sixty phosphopeptides were identified and quantified in both biological replicates using quantitative liquid chromatography-tandem mass spectrometry (Fig. 2A and B). Ten phosphopeptides had a fold change ratio larger than 1.5-fold or smaller than 0.67-fold in both biological replicates; interexperiment variation was low for six phosphopeptides (coefficient of variation < 30%) (Fig. 2B; Table S2 and Data Set S1). We selected a phosphopeptide identified in the actin-binding protein EhCoactosin (EHI_168340) for follow-up studies, because EhCoactosin was previously implicated in E. histolytica virulence and is believed to function in the control of actin dynamics (19, 20). The EhCoactosin S147-containing phosphopeptide (EHI_168340A) AGGADYSFNTTS(phospho)N showed an average fold change of 1.93 (*EhMSP-1-*silenced/control) (see Table S2). The measured precursor mass of the identified peptides was within 1 ppm of the theoretical mass and the tandem mass spectrometry (MS/MS) spectrum exhibited a continuous stretch of b or y ion series with clear peak assignments, indicating confident identification of the coactosin phosphopeptide (Fig. 3). The absence of any significant change in EhCoactosin abundance after gene silencing (Fig. 1B) suggested that the increased abundance of phosphopeptide EhCoactosin S147 could be due to altered cell signaling in the EhMSP-1-deficient cells.

10.1128/mBio.00660-20.6 TABLE S2 Phosphopeptides with a fold change either >1.5-fold or <0.67-fold (coefficient of variation [CV] < 30%) following *EhMSP-1* silencing. Download Table S2, PDF file, 0.1 MB. Copyright © 2020 Hasan et al. 2020 Hasan et al. This content is distributed under the terms of the Creative Commons Attribution 4.0 International license.

### Cellular localization of EhCoactosin does not change with phosphomimetic and phosphorylation-deficient mutations.

Coactosin is a member of the ADF/cofilin family of actin-binding proteins (24). Recombinant EhCoactosin was previously shown to interact directly with F-actin, and the two proteins colocalize during E. histolytica phagocytosis of host cells (20). Another study reported that EhCoactosin is overexpressed upon pharmacological inhibition of amoebic phosphatidylinositol 3-kinase (19). Although the related ADF protein cofilin is phosphoregulated, coactosin itself is not known to be phosphoregulated.

**FIG 4:**
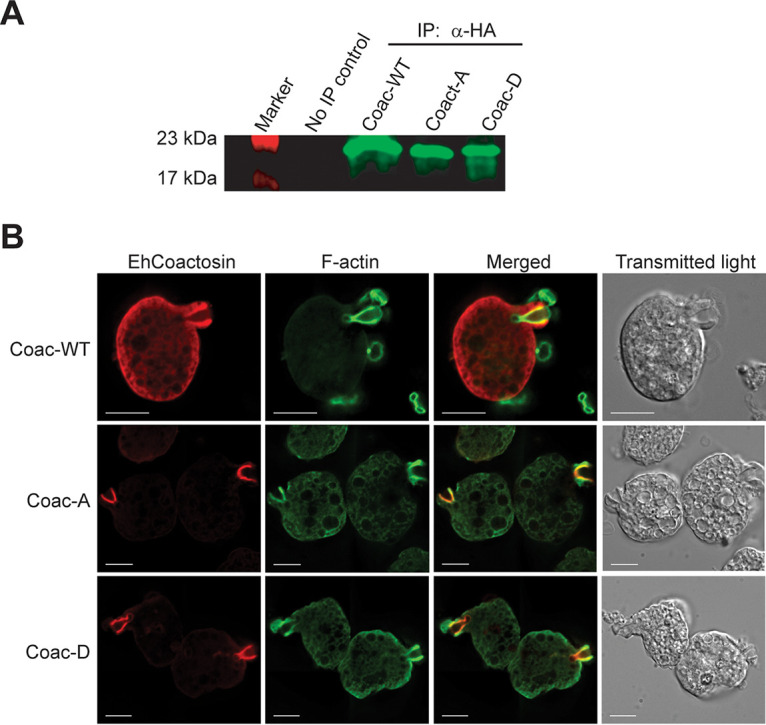
Expression and localization of nonphosphorylatable (S147A) and phosphomimetic (S147D) EhCoactosin variants. HM-1:IMSS strain trophozoites were transfected with expression vectors designed for tetracycline-regulated EhCoactosin expression with an N-terminal hemagglutinin (HA) epitope tag fused to either wild type (Coac-WT), S147A (Coac-A), or S147D (Coac-D). (A) Western blot. Protein expression was induced with tetracycline followed by immunoprecipitation (IP) and blotting with an anti-HA antibody. The expected protein size was 17 kDa. (B) Localization of EhCoactosin and S147A and S147D variants during erythrophagocytosis. Trophozoites expressing each EhCoactosin variant protein were incubated with human erythrocytes and then prepared for fluorescence microscopy by staining F-actin with phalloidin (green) and the EhCoactosin proteins with a mouse anti-HA monoclonal antibody. The Coac-WT, Coac-A, and Coac-D proteins all localized to the phagocytic cup. Bars, 10 μm.

To test the physiologic relevance of EhCoactosin phosphorylation, we expressed three variants of it with an N-terminal hemagglutinin (HA) tag under the control of a tetracycline-inducible promoter in HM-1:IMSS strain trophozoites. The following tagged proteins were expressed: EhCoactosin wild type (Coac-WT), EhCoactosin S147D (Coac-D; phosphomimetic mutation), and EhCoactosin S147A (Coac-A; phosphorylation-deficient mutation). Western blotting performed following anti-HA immunoprecipitation verified expression of the full-length proteins (Fig. 4A). We next assessed the localization of each EhCoactosin variant and F-actin *in vivo* using immunofluorescence microscopy. EhCoactosin showed a general cytoplasmic staining pattern, occasionally colocalizing with F-actin. The overall pattern did not vary between the three EhCoactosin variants. When actin polymerization was stimulated by forcing the interaction of trophozoites with erythrocytes, all three variants colocalized with F-actin within phagocytic cups (Fig. 4B).

### Overexpression of EhCoactosin mutants affects trophozoite membrane protrusions and motility.

**FIG 5:**
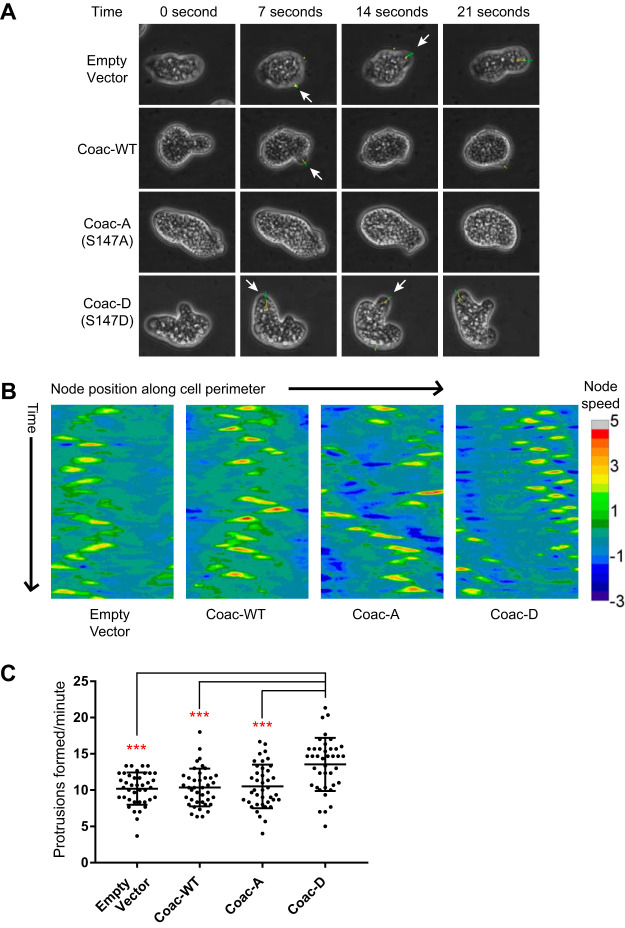
Phosphomimetic (S147D) EhCoactosin expression increased the formation of E. histolytica cell membrane protrusions. Qualitative differences in appearance of the empty vector control-, wild-type (Coac-WT)-, S147A (Coac-A)-, and S147D (Coac-D)-expressing trophozoites in culture were quantified using the NIH ImageJ plugin QuimP3 and time-lapse light microscopy. (A) Representative time-lapse images for coactosin-overexpressing and vector control trophozoites. An increased rate of pseudopod formation in the Coac-D-expressing trophozoites was immediately evident in tissue culture (see also Movies S1 and S2). Time is set as 0 s for the first image of the presented series. Yellow-green lines denote membrane protrusion sites identified by QuimP3. Regions of cell membrane scored by QuimP3 are highlighted by program-generated lines color-coded according to the rate of expansion (as in panel B). For clarity, these areas are indicated with arrows at the time of first appearance (i.e., one arrow per pseudopod scored by QuimP3). (B) Motility maps generated by QuimP3 for video microscopy of representative empty vector, Coac-WT, Coac-A, and Coac-D trophozoites. The *x* axis denotes the normalized position of 1,200 defined nodes along the cell perimeter, and the *y* axis denotes the video frame number (i.e., time). The color scale corresponds to membrane velocity at each point along the cell perimeter at a given time. Cell protrusions are identified as sites of maximum local velocity. (C) Effect of EhCoactosin S147A and S147D expression on the number of cell membrane protrusions versus time. Data for the empty vector, Coac-WT, Coac-A, and Coac-D strains are shown. Cells (*n* = 40 per cell line) were imaged for 3 min with 0.4-s intervals, and cell protrusions were identified with QuimP3. Data shown are the number of cell protrusions formed/minute by each analyzed amoeba, along with the mean and standard deviation (SD) for each cell line. ***, *P* < 0.0001 for ordinary one-way analysis of variance (ANOVA) with Tukey’s multiple-comparison test.

Despite the absence of major differences in interaction with F-actin and protein localization, it was immediately evident following the selection of stable transfectants that the EhCoactosin mutant-expressing trophozoite strains appeared different in culture; specifically, Coac-D-overexpressing trophozoites seemed to have an increased number of small cell membrane protrusions but to migrate less efficiently than Coac-A-overexpressing cells. On the other hand, the more motile Coac-A-expressing strain had fewer but larger pseudopods (Fig. 5A). To quantify the differences in membrane protrusion, we utilized NIH ImageJ software and a previously described cell protrusion analysis plugin called QuimP (25). We captured time-lapse images of trophozoites on a glass surface for 3 min (see Movies S1 and S2) and then analyzed individual trophozoites with QuimP. One of the outputs of the plugin is called a “motility map,” which allows the detection of membrane protrusions by plotting directional velocities of cell membranes as pixel values across time. Figure 5B shows example motility maps from individual empty vector control-, Coac-WT-, Coac-A-, and Coac-D-expressing cells. The motility maps suggested that Coac-D-expressing cells generated more patches of high pixel values than the others, indicating a greater number of regions of extending cell membrane. To quantify this phenotype, we measured the number of protrusions detected by the plugin for multiple cells (see Movie S2). As expected based on the observations in culture, Coac-D cells formed significantly more membrane protrusions than Coac-A, Coac-WT, and empty vector control trophozoites (Fig. 5C).

10.1128/mBio.00660-20.1 MOVIE S1 Time-lapse images of coactosin overexpression mutants and vector control trophozoites. Fields of each of the trophozoite lines on glass coverslips were imaged for 3 min with 0.4-s intervals. Fifteen frames per second .avi format videos were prepared using ImageJ. Download Movie S1, AVI file, 19.0 MB. Copyright © 2020 Hasan et al. 2020 Hasan et al. This content is distributed under the terms of the Creative Commons Attribution 4.0 International license.

10.1128/mBio.00660-20.2 MOVIE S2 Protrusion tracking of a representative trophozoite for each E. histolytica cell line. Protrusion tracking output with protrusion analysis algorithm of ImageJ plugin QuimP. Original images were captured for a duration of 3 min with 0.4-s intervals. Tracks of detected protrusions were overlaid on original time-lapse images. Fifteen frames per second .avi format videos were prepared using ImageJ. Download Movie S2, AVI file, 17.7 MB. Copyright © 2020 Hasan et al. 2020 Hasan et al. This content is distributed under the terms of the Creative Commons Attribution 4.0 International license.

10.1128/mBio.00660-20.3 MOVIE S3 Manual tracking of a representative trophozoite for each E. histolytica cell line. (Left) Time lapse of a trophozoite from the labeled mutant line in Matrigel captured for a duration of 2 h with 2-min intervals. (Right) Motility track of the same trophozoite prepared using the manual tracking plugin of ImageJ. Original image and motility tracks were combined and 3 frames per s .avi format videos were prepared using ImageJ. Download Movie S3, AVI file, 11.0 MB. Copyright © 2020 Hasan et al. 2020 Hasan et al. This content is distributed under the terms of the Creative Commons Attribution 4.0 International license.

**FIG 6:**
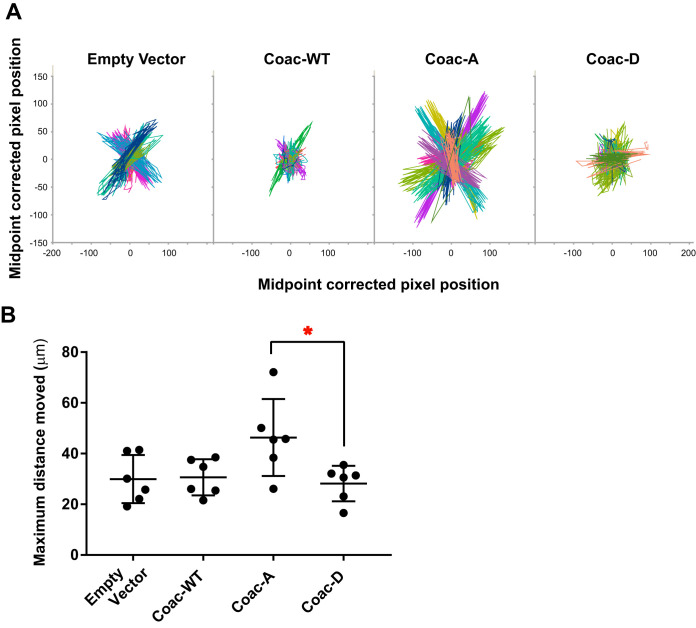
EhCoactosin S147A expression increases the range of E. histolytica movement within a model extracellular matrix. Trophozoites trapped within Matrigel matrix were imaged for 2 h at 2-min intervals, and movement of individual trophozoites was tracked using the manual tracking plugin of NIH ImageJ. Six independent experiments were conducted with the empty vector-, wild type (Coac-WT)-, EhCoactosin S147A (Coac-A)-, and EhCoactosin S147D (Coac-D)-expressing trophozoites, with 18 trophozoites imaged/cell line/experiment. (A) Superimposed motility tracks for the vector control and EhCoactosin WT-, S147A (Coac-A)-, and S147D (Coac-D)-overexpressing trophozoites. Tracks from one experiment are shown (*n* = 18 per cell line). Pixel coordinates for each trophozoite from the original images were corrected to the midpoints of the extreme *x* and *y* axis values for display on a single graph. Pixel length = 0.32 μM. (B) Effect of EhCoactosin S147A and S147D expression on amoebic movement. The distance between the two farthest points was determined for each trophozoite and used to determine the mean range of movement (i.e., maximum distance moved) for each cell line during a given experiment. Each data point represents the mean maximum distance moved for the 18 trophozoites of that cell line measured in one experiment, with data for six biological replicates shown. *, *P* < 0.03 by one-way ANOVA with Tukey’s multiple-comparison test.

Cell motility was the other phenotype of primary interest. During invasive amoebiasis, the motility of trophozoites comes into play when they move through the ECM-rich colonic tissue layers. To mimic that complex 3-dimensional (3D) environment, we studied parasite motility within Matrigel, a commercially available model of mammalian ECM. Initially, E. histolytica did not survive long enough in Matrigel to quantify the motility of a large number of trophozoites, but we found empirically that addition of Escherichia coli (strain DH5α) to the Matrigel preparation prolonged E. histolytica survival. Time-lapse images of each EhCoactosin mutant-overexpressing cell line moving within Matrigel were used to generate motility tracks of individual cells with the manual cell tracking plugin of NIH ImageJ (https://ImageJ.nih.gov/ij/plugins/track/track.html) (Movie S3). Note that this method assessed nondirectional movement of the trophozoites, which generally moved back and forth within the same region. Consistent with the appearance of these cells in tissue culture, the motility tracks indicated that the Coac-A trophozoites had larger motility zones (Fig. 6A). We calculated the distance between the two farthest points of every trophozoite’s motility track to quantify this phenotype. The results confirmed that Coac-A-expressing amoebae moved significantly further than Coac-D-expressing cells in this 3D environment (Fig. 6B).

### Coactosin overexpression mutants do not differ in adherence and phagocytosis.

**FIG 7:**
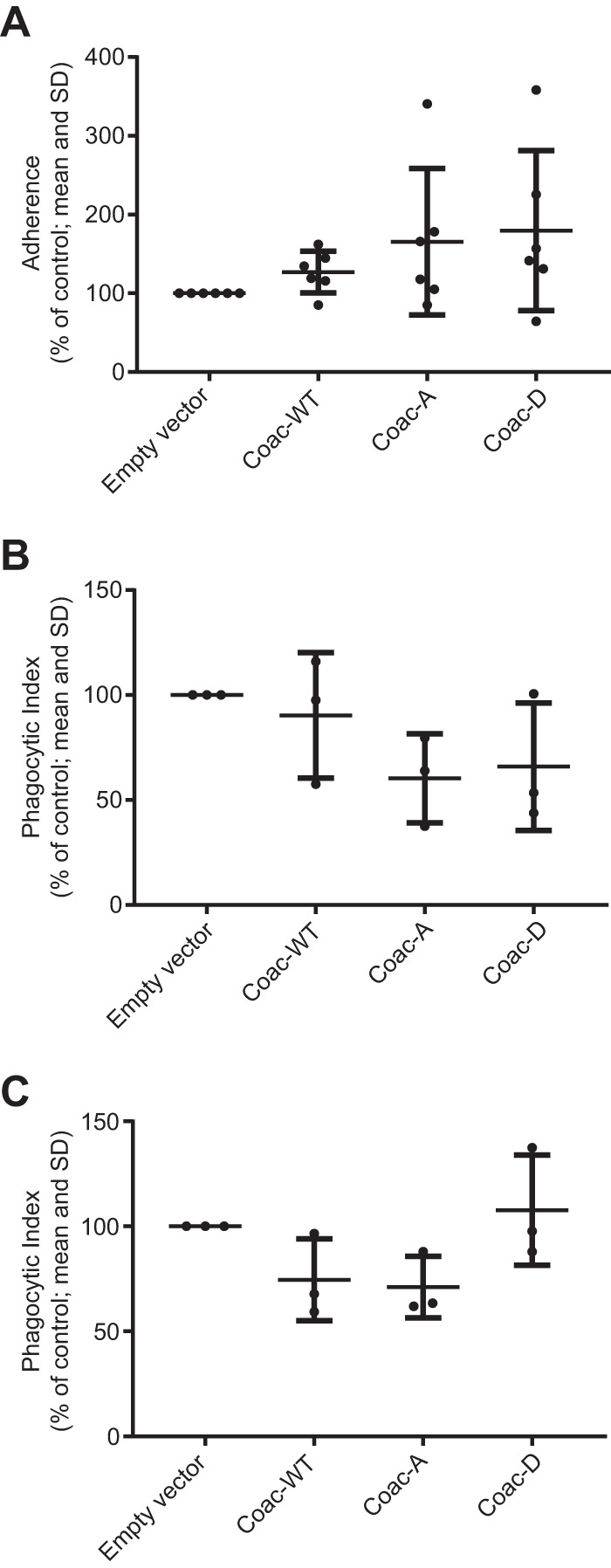
EhCoactosin S147A and S147D do not affect E. histolytica adherence or phagocytosis. (A) Adherence to fixed Chinese hamster ovary (CHO) cells. To enable combined analysis of multiple experiments, the number of adherent trophozoites for each cell line was expressed as the percentage of adherent empty vector control cells adherent on that day (*n* = 6 independent experiments). Lines indicate means and SDs. There was considerable variability from day to day. Expression of the Coac-WT, Coac-A, and Coac-D variant EhCoactosin proteins had no significant effect. Phagocytosis of healthy (B) and apoptotic (C) Jurkat T lymphocytes. Phagocytosis of fluorescently labeled Jurkat cells was quantified for each trophozoite line using flow cytometry, and the phagocytic index was calculated as the percentage of phagocytic amoebae × the mean fluorescence index of phagocytic amoebae. See Fig. S1 in the supplemental material for the flow cytometry gating scheme. The means from multiple readings in three independent experiments were expressed as the percentage of phagocytic index for empty vector control cells to enable combined statistical analysis for experiments performed on multiple days. Each data point indicates the average normalized phagocytic index for 1 day (*n* = 3 for each mutant). Expression of the Coac-WT, Coac-A, and Coac-D variant EhCoactosin proteins had no significant effect.

Cell membrane protrusion is linked to numerous biological processes in addition to cell motility, including adherence and phagocytosis (26, 27), both of which are correlated with amoebic virulence. We therefore tested the effects of EhCoactosin mutant overexpression on adherence and phagocytosis. Adherence is coupled to membrane protrusion mainly via actin dynamics and signaling pathways. We used a standard E. histolytica adherence assay, which measures amoebic adherence to glutaraldehyde-fixed Chinese hamster ovary (CHO) cell monolayers. Surprisingly, the EhCoactosin overexpression mutants exhibited no significant differences in adherence (Fig. 7A). Similarly, despite clear localization of each of the EhCoactosin mutant proteins to the phagocytic cup, overexpression of the EhCoactosin mutant proteins did not alter the parasite’s ability to phagocytose healthy host cells (Fig. 7B). Furthermore, since cell killing appears to be the rate-limiting step in rapid phagocytosis of viable cells (28), we assayed phagocytosis of apoptotic cells and again found no significant difference (Fig. 7C).

10.1128/mBio.00660-20.7 FIG S1 Flow cytometry-based phagocytosis assay. Target cells (viable or UV-killed Jurkat lymphocytes) are fluorescently stained with CFSE and incubated with E. histolytica trophozoites at 37°C. The samples are then analyzed by flow cytometry. Amoebas are distinguished from lymphocytes using forward scatter (FSC) and side scatter (SSC) characteristics, and phagocytic amoebae are defined as those with fluorescence levels greater than baseline. Data are then expressed as a phagocytic index, calculated as the mean fluorescence of phagocytic trophozoites × the percentage of phagocytic trophozoites, and normalized to the empty vector control to enable combined analysis of experiments performed on different days. The figure shows example data. (A) SSC versus FSC for control sample with E. histolytica trophozoites alone. (B) SSC versus FSC for a sample with viable CFSE-labeled Jurkat lymphocytes alone. The gate shown in panels A and B identifies the majority of trophozoites while excluding nonphagocytosed host cells. (C) Example fluorescence histograms for amoebas identified by the gate in panels A and B for amoebas incubated alone and for each cell line incubated with CFSE-labeled Jurkat cells, shown either as an overlay on the left or as aligned histograms on the right. The M1 gate identifies nonphagocytic trophozoites, and the M2 gate identifies phagocytic trophozoites. Red, amoeba baseline fluorescence (i.e., no labeled lymphocytes); blue, empty vector control; orange, Coac-WT; light green, Coac-A; dark green, Coac-D. Download FIG S1, PDF file, 0.2 MB. Copyright © 2020 Hasan et al. 2020 Hasan et al. This content is distributed under the terms of the Creative Commons Attribution 4.0 International license.

## DISCUSSION

To better understand the mechanism of previously observed changes in E. histolytica adherence, motility, and invadosome formation after silencing expression of the metallosurface protease EhMSP-1 (11, 14), we used a combination of two affinity enrichment methods (22, 23) and mass spectrometry to identify phosphopeptides with altered abundance in *EhMSP-1-*silenced versus control E. histolytica trophozoites. This analysis identified six phosphopeptides with either increased or reduced abundance after *EhMSP-1* silencing, including phosphopeptides in an unconventional myosin IB and EhCoactosin, two proteins known to function in control of actin dynamics. In mammals, the ADF/cofilin family protein coactosin-like protein 1 (Cotl1) competes with cofilin for F-actin binding and functions in the control of motility at the leading edge of migrating cells. Given this and the well-described phosphoregulation of cofilin (Cotl1 is not reported to be phosphoregulated), we focused follow-up studies on EhCoactosin. Regulated overexpression of EhCoactosin variants with phosphomimetic and nonphosphorylatable substitutions at S147 confirmed that S147 phosphorylation of EhCoactosin functions in coordination of amoebic cell membrane dynamics and motility during migration through a tissue matrix. These studies also validated the phosphoproteomics data set.

EhMSP-1 is neither a kinase nor a phosphatase, and our study does not address if EhMSP-1 functions directly in outside-to-in ECM sensing by E. histolytica; indeed, the changes seen following *EhMSP-1* silencing could be a result of compensatory changes. However, EhMSP-1 is a leishmanolysin-like M8 family metalloendopeptidase (14). The substrates of homologous M8 family metalloendopeptidases are frequently ECM components or cell surface proteins, and such proteases often participate in both outside-to-inside and inside-to-outside signaling pathways that are frequently parts of protein kinase-phosphatase signaling cascades (29–32). Interestingly, the *Drosophila* M8 family metalloendopeptidase invadolysin localizes to invadosomes and, among many other tasks, functions in the migration of germ cells through tissue (33); this is particularly interesting given the previously reported altered adherence, motility, and invadosome formation of *EhMSP-1*-silenced E. histolytica trophozoites (14). Taken together with the fact that *EhMSP-1* silencing affects trophozoite adhesion and motility (14), i.e., cellular processes known to be dynamically regulated by protein phosphorylation, it is not surprising that we identified altered protein phosphorylation in the *EhMSP-1-*silenced and control E. histolytica lines. Further studies are needed to determine the function of EhMSP-1 and if it participates in amoebic degradation and sensing of the extracellular matrix.

If we consider the motility phenotypes of the *EhMSP-1*-silenced line (14) and EhCoactosin Coac-A and Coac-D overexpression lines, we see that they agree. *EhMSP-1*-silenced mutants are less motile and more frequently phosphorylated at S147 of EhCoactosin. Similarly, trophozoites overexpressing the phosphomimetic mutant of EhCoactosin are less motile. This suggests that EhCoactosin dephosphorylation may occur downstream of an action of EhMSP-1 during trophozoite movement, but as noted above, our study does not directly address if EhMSP-1 functions in signaling.

A published crystal structure and *in vitro* actin-binding assays using a truncated form of EhCoactosin demonstrated that EhCoactosin binds to both F- and G-actin and becomes enriched at sites of E. histolytica actin polymerization (e.g., erythrophagocytic cups) (20). Here, we showed that EhCoactosin was more frequently phosphorylated at S147 following *EhMSP-1* silencing, an amino acid not included in the solved crystal structure. Additional EhCoactosin phosphorylation sites cannot be fully excluded, but our affinity enrichment and mass spectrometry experiments detected no additional sites. The increased abundance of S147-phosphorylated EhCoactosin could not be attributed to an overall increase in EhCoactosin abundance, suggesting a functional role of EhCoactosin phosphorylation in controlling the protein’s effects on actin dynamics. In a separate study, EhCoactosin was identified as being involved directly or indirectly in the amoebic phosphatidylinositol 3-kinase (PI3K) pathway (19).

The mammalian homologue of EhCoactosin is called coactosin-like protein 1 (Cotl1), and there are several studies confirming its involvement in cell migration and membrane protrusion (24, 34–37). For example, overexpression of mouse Cotl1 impairs the characteristic migration of neurons during cerebral cortex development (36), and depletion of human Cotl1 impairs lamellipodial formation in Jurkat T lymphocytes (35). (35, 38, 39). Note that S147 is not a conserved residue in most model eukaryotes or in mammals (see Fig. S2 in the supplemental material). However, there is mass spectrometry evidence of phosphorylation at the penultimate residue in mammalian Cotl1 (T141 in human and S141 in mouse and rat) (40), suggesting that Cotl1 may also be phosphoregulated. Furthermore, multiple-sequence alignment reveals that S147 of EhCoactosin is a conserved residue in other *Entamoeba* species, as well as in a few other species of the Amoebozoa lineage (Fig. S2). The EhCoactosin S147 phosphorylation and regulatory mechanism may therefore be conserved in this group of organisms, including in some nonpathogenic *Entamoeba* species.

10.1128/mBio.00660-20.8 FIG S2 Multiple-sequence alignment of coactosin homologs from selected species. The species names are indicated on the left. The E. histolytica coactosin row is marked with a red border, and the amino acid position corresponding to S147 of EhCoactosin is marked with a black box and an asterisk. Download FIG S2, PDF file, 0.7 MB. Copyright © 2020 Hasan et al. 2020 Hasan et al. This content is distributed under the terms of the Creative Commons Attribution 4.0 International license.

Cotl1 competes for F-actin binding with cofilin, a phosphoregulated protein that plays a key role at the leading edge of motile cells. Cofilin severs F-actin and thereby creates new actin barbed ends for actin polymerization while also degrading old actin filaments (16–18). Regulation of cofilin activity is highly complex and dependent on the balance of inactive (i.e., phosphorylated and sequestered) versus active (i.e., dephosphorylated and free to bind actin) cofilin (16). For example, phosphorylated cofilin is sequestered by 14-3-3 and thus maintained as inactive (70). Upon stimulation with chemoattractants, some motile cells such as neutrophils dephosphorylate cofilin, thus releasing it to bind F-actin (41, 42). In contrast, migrating cancer cells appear to maintain the majority of cofilin in a dephosphorylated state and may respond to stimuli through the phosphorylation of cofilin (42). LIM kinase (LIMK) and TES kinase (TESK) are both known to phosphorylate cofilin, and the dephosphorylated cofilin pool is replenished by a variety of cofilin phosphatases, including protein phosphatase 1 (PP1), protein phosphatase 2A (PP2A), and slingshot (SSH) (16, 17, 43, 44). The extent to which these regulatory mechanisms are conserved among Amoebozoa is a key area for future investigation, and given the role of cell migration in the pathogenesis of invasive amoebiasis, identification of the relevant kinases and phosphatases may yield novel therapeutic targets.

We tested the effect of the EhCoactosin phosphorylation on trophozoites by regulated episomal overexpression of phosphomimetic and phosphorylation-deficient variants of EhCoactosin. Although this method is state of the art for *Entamoeba* research (allelic exchange has not yet been successful in E. histolytica), it is limited by potential confounding effects from the unaltered genomic copy of the EhCoactosin gene. The extent to which presence of wild-type EhCoactosin masked the phenotypes quantified following overexpression of the S147D and S147A EhCoactosin variants is unclear, but it is likely that its presence reduces the effects of EhCoactosin mutant expression. As discussed later, we performed several phenotypic assays using these cell lines for which no differences were detected. Given the limitations of the experimental system, we cannot conclude that these phenotypes are unaffected by EhCoactosin phosphorylation; rather, we conclude from the mutant protein overexpression studies that EhCoactosin phosphorylation is functionally relevant for the control of cell membrane dynamics and cell migration.

The most easily detectable localization of EhCoactosin in trophozoites is with F-actin within phagocytic cups. Since Coac-A and Coac-D both colocalized with F-actin during phagocytosis, it appears that EhCoactosin phosphorylation does not affect the F-actin binding capacity of EhCoactosin. Previous work indicates that EhCoactosin stabilizes F-actin (20). Therefore, EhCoactosin phosphorylation might have its effect by changing its ability to alter actin dynamics upon F-actin binding. EhCoactosin can also bind to G-actin, and truncating the C-terminal of EhCoactosin slightly decreases its affinity to G-actin (20). G-actin-binding proteins can alter the actin dynamics of the cell either by sequestering or recruiting actin monomers to F-actin. Actin binding and polymerization/depolymerization assays (described in reference 20) using recombinant EhCoactosin S147A and EhCoactosin S147D would provide additional insight into how EhCoactosin phosphorylation alters amoebic actin dynamics.

The general idea that we formed by observing cultures of trophozoites after inducing protein expression with tetracycline was that the Coac-D trophozoites formed more cell membrane protrusions but protrusions that were smaller in size and duration. The NIH ImageJ plugin QuimP allowed us to quantify this phenotype. While expression of the Coac-D mutant protein resulted in significantly more protrusions, the lifetimes of the protrusions were not significantly different. Visual inspection of the motility maps suggested that the protrusions were indeed smaller following Coac-D overexpression. Depending on cell type, F-actin structure and dynamics, and involvement of various actin-binding proteins, membrane protrusions can be divided into several types, including lamellipodia, filopodia, pseudopods, invadosomes, and blebs (45–47). As we performed our protrusion tracking using transmitted light images, these protrusion subtypes could not be distinguished.

The motility assay presented here tests the movement capability of trophozoites in a direction-independent manner. We envision that integrating any trophozoite chemoattractant or chemorepellent molecule in the system would greatly increase the power of this assay, especially based on reported effects of chemoattractants on cofilin phosphorylation and dephosphorylation in mammalian cells (41–43). Unfortunately, our attempts to do this using previously reported compounds were unsuccessful (48, 49). Additionally, there are published experiments of testing the invasive capability of E. histolytica using colon tissue explants and animal models (50–54). Testing our EhCoactosin overexpression mutants in those experimental setups would be useful to determine the clinical significance of our observed motility phenotype.

The Coac-A mutant is significantly more motile in Matrigel than the Coac-D-expressing cell line, and the pseudopod formation phenotype provides a clue to the likely mechanism underlying this observation. The Coac-D mutant forms a significantly greater number of pseudopods than the other trophozoite lines tested in this study. This may initially appear to conflict with the observation that the Coac-D mutant moves less efficiently, but on reflection, it suggests that EhCoactosin phosphoregulation likely plays a critical role in coordinating pseudopod growth and retraction. Consistent with this, the pseudopods formed by Coac-D-expressing amoebae were both different in number and qualitatively different from those formed by the other trophozoite lines. Perhaps this aberrant pseudopod formation interferes with the coordination of trophozoite motility.

An additional interesting observation made while establishing the 3D motility assay used here is that E. histolytica trophozoites remain viable in Matrigel longer in the presence of E. coli. Based on publications prior to growth of E. histolytica in axenic culture, an obvious potential explanation is simply that the bacteria prolong amebic survival by providing nutrition (55). However, it is now well established that E. histolytica infection is impacted by the intestinal microbiome and that bacteria alter E. histolytica cell signaling and transcriptomics (4, 56–58). Thus, the effect of E. coli in this Matrigel environment could potentially regulate cellular processes relevant for amoebic migration and virulence and presents an avenue for further investigation.

Given the known roles of actin polymerization in amoebic adherence and phagocytosis and that EhCoactosin localizes within phagocytic cups and its overexpression reduced phagocytosis by ∼50% in a prior report (20), it is somewhat surprising that adherence and phagocytosis were not significantly affected by Coac-A and Coac-D overexpression. Furthermore, although cell killing was not directly assayed, we note that cell killing is the rate-limiting step in phagocytosis (28); thus, the absence of an effect on phagocytosis of viable cells suggests that cell killing is also unaffected by expression of these protein constructs. As noted above, a limitation of this experimental system is the ongoing expression of the genomic copy of the EhCoactosin gene, which might cause false-negative results. We also note that these assays are limited by considerable day-to-day variability. Indeed, our data show small but statistically nonsignificant trends toward altered adherence and phagocytosis, but even if real, we believe that any such effect would be small and of unclear biological significance.

In addition to continued efforts to identify kinases, phosphatases, and upstream mechanisms controlling EhCoactosin phosphorylation, follow-up experiments on other differentially phosphorylated proteins identified in this study would be of interest. The unconventional myosin IB (EHI_110810) is consistently hypophosphorylated in *EhMSP-1-*silenced cells. This unconventional myosin has the characteristic structural organization of myosin 1 family proteins (i.e., a head domain having motor function, a neck domain having a light-chain-binding domain, and a tail containing TH1 [for binding phospholipids], TH2 [for binding other proteins], and SH3 signaling domains) (59–61). The identified phosphorylation site falls within the putative TH2 domain, suggesting that this phosphorylation site might function in regulating the membrane localization or phosphoinositide signaling of myosin IB. Overexpression of myosin IB reduced the rate of erythrocyte phagocytosis (61, 62). Furthermore, the calcium binding proteins EhCaBP3 and EhCaBP5 and the Rho GTPase exchange factor EhFP10 all interact with this unconventional myosin IB (63). Therefore, phosphoregulation of EHI_110810 might impact multiple E. histolytica virulence properties.

Finally, note that in order to specifically identify differentially phosphorylated proteins, we determined overall protein abundance in *EhMSP-1-*silenced and vector control trophozoites. Normalization for protein abundance served as a critical control to ensure that proteins believed to be differentially phosphorylated in these cell lines did not simply have altered abundance. Nonetheless, proteins with altered abundance in the *EhMSP-1*-silenced and control cell lines were identified, and while compensatory, these changes (see Table S1) may be involved in the phenotypic differences between these cell lines and of interest to the *Entamoeba* research community.

Entamoeba histolytica remains an important cause of severe diarrhea and liver abscess in developing countries, and additional work is needed to understand what determines amoebic tissue invasiveness. These data demonstrate differential protein phosphorylation in amoebic cell lines with *EhMSP-1-*silenced versus control cells, which were previously shown to have altered adherence, motility, and ability to form invadosomes associated with ECM degradation. Validating the phosphoproteomics data set, follow-up studies on EhCoactosin show that differential phosphorylation of S147 functions in control of E. histolytica cell membrane dynamics and amoebic motility within a tissue matrix. Future studies using the substrates identified here will be focused on identifying the kinases and phosphatases involved in the control of amoebic invasiveness in hopes of providing mechanistic insight and potentially new drug targets.

## MATERIALS AND METHODS

### Cell cultures.

Entamoeba histolytica HM-1:IMSS or G3 strain trophozoites were cultivated axenically in TYI-S-33 medium (64). For phagocytosis and adherence assays, trophozoite cultures were harvested after 48 h (log-phase growth) by chilling on ice and resuspended in M199s (M199 medium supplemented with 25 mM HEPES [pH 6.8], 5.7 mM cysteine, and 0.5% bovine serum albumin [BSA]).

*EhMSP-1* was silenced in HM-1:IMSS and G3 strain trophozoites using previously described methods (14, 65, 66). Both *EhMSP-1*-silenced strains and control cells (empty vector-transfected parasites) were grown in TYI-S-33 medium with 30 μg/ml G418 selection over a period of 4 weeks and then grown without selection. Chinese hamster ovary (CHO) cells were grown to confluence on 24-well plates in F-12K medium containing l-glutamine with 10% fetal bovine serum (FBS), 200 U/ml penicillin G, and 200 μg/ml streptomycin sulfate. Jurkat T lymphocytes (clone E-6) were cultured in RPMI 1640 medium with 10% FBS, 200 U/ml penicillin G, and 200 μg/ml streptomycin sulfate. Human red blood cells (HRBC) used were from expired units of packed red blood cells provided by the University of Vermont Medical Center blood bank.

### Quantitative proteomics and phosphoproteomics.

See Text S1 in the supplemental material for detailed methods. G3 strain empty vector control and *EhMSP-1-*silenced [EhMSP-1 (−)] trophozoites were used for all proteomics and phosphoproteomics experiments. For quantitative proteomics, cell pellets were reconstituted in urea with phosphatase inhibitors (Halt phosphatase inhibitor cocktail) and sonicated, and 20 mg of protein was digested with trypsin. Tryptic peptides were purified, lyophilized, labeled with stable isotopes by dimethyl labeling, and then cleaned by Sep-Pak tC_18_ column. An aliquot of each sample was used for total protein expression profiling by liquid chromatography-mass spectrometry (LC/MS) analysis, and the remainder of each sample was lyophilized for subsequent phosphopeptide enrichment. A two-step procedure was used to enrich phosphopeptides for quantitative phosphoproteomics. First, purified labeled peptides were reconstituted in 0.1% trifluoroacetic acid (TFA) 50% acetonitrile (CH_3_CN) and incubated with a slurry of immobilized metal affinity chromatography (IMAC) beads (PHOS-Select iron affinity gel, 10 mg of peptides/ml 50% slurry; Sigma), and bound phosphopeptides were eluted twice with 10× bead volume 1% NH_4_OH (pH 11). Second, the unbound fraction of peptide solution was subjected to further phosphopeptide enrichment using TiO_2_ beads (GL Sciences, Tokyo, Japan) and then eluted sequentially with 400 μl 5% NH_4_OH (pH 11) and 400 μl 5% pyrrolidine aqueous solution. All eluates were then dried down, purified by STAGE tips or Ziptips, reconstituted with 2.5% CH_3_CN-2.5% formic acid (FA), and analyzed by nanoscale LC/MS. Peptides were identified using the SEQUEST search engines Proteome Discoverer 2.2 (Thermo Fisher, Waltham, MA) and the AmoebaDB database.

10.1128/mBio.00660-20.4 TEXT S1 Detailed methods for quantitative phoshoproteomics and video microscopy analysis using QuimP3 for identification of pseudopods. Download Text S1, PDF file, 0.1 MB. Copyright © 2020 Hasan et al. 2020 Hasan et al. This content is distributed under the terms of the Creative Commons Attribution 4.0 International license.

### Preparation of EhCoactosin-overexpressing trophozoite lines.

The EhCoactosin gene was amplified from E. histolytica genomic DNA and cloned between KpnI and BamHI sites of a Ptet plasmid, which puts the transgene under a tetracycline-inducible promoter and carries a constitutively expressed hygromycin resistance gene (67, 68). We introduced an HA tag in the upstream primer (5′-CGGGGTACCATGTATCCATATGATGTTCCAGATTATGCTATGTCTGGATTTGATCTT-3′). The desired mutations were introduced into the downstream primer. The primers were 5′-GCGGGATCCTTAATTTGAGGTGGTATTGAAAGAGTAATCAGC-3′ for wild-type EhCoactosin, 5′-GCGGGATCCTTAATTAGCGGTGGTATTGAAAGAGTAATCAGC-3′ for EhCoactosin S147A, and 5′-GCGGGATCCTTAATTATCGGTGGTATTGAAAGAGTAATCAGC-3′ for EhCoactosin S147D. HM-1:IMSS trophozoites were transfected with these plasmids or an empty-vector control plasmid using the transfection reagent Attractene (no. 301005; Qiagen), and transfectants were selected with hygromycin with the dose increased each week until reaching a maintenance dose of 15 μg/ml (68).

### Immunofluorescence microscopy and erythrophagocytosis.

Suspensions of 2.0 × 10^5^ amoeba trophozoites were added to 4.0 × 10^5^ HRBC in a final volume of 150 μl M199s medium, distributed onto uncoated 35-mm glass-bottom culture dishes (MatTek 14 mm microwell, 1.5 cover glass, 0.16 to 0.19 mm), and incubated for 15 min at 37°C to allow amoebic phagocytosis. Nonadherent cells were aspirated off the plate, while adhered cells were fixed with 1.5 ml 4% formaldehyde for 20 min at room temperature (RT) and then permeabilized with 100 μl 0.2% Triton X-100 for 3 min. Cells were washed twice with 2 ml phosphate-buffered saline (PBS), blocked with 1.5% BSA-PBS for 30 min at RT, washed twice, and incubated for 1 h at RT with mouse monoclonal anti-HA antibody (clone HA-7; Sigma) (2.0 μg/ml) diluted in blocking buffer. After incubation, cells were washed with 2.0 ml PBS and stained for 1 h at RT with 100 μl goat anti-mouse IgG Alexa Fluor 568-conjugated antibody (Invitrogen) (10 μg/ml in blocking buffer). Cells were then washed and incubated for 1 h at RT with 100 μl Alexa Fluor 488-labeled phalloidin (Invitrogen) at 1:40 dilution in PBS. Cells were washed twice with 2.0 ml PBS, resuspended in 2.5 ml 0.02% sodium azide-PBS, and visualized using a 60× oil immersion lens objective (1.4 numerical aperture [NA]).

### Quantifying E. histolytica cell membrane protrusions.

To count the rate of formation of cell membrane protrusions by trophozoites, we induced EhCoactosin overexpression by treating cultures with 5 μg/ml tetracycline for 24 h before resuspending trophozoites in 2 ml complete TYI. Trophozoites were then allowed to settle for 10 min onto MatTek glass-bottom dishes before imaging. We captured transmitted light time-lapse images for 3 min with 0.4-s intervals using a 20× lens objective (0.5 NA). Images were exported in .tiff format, opened as image sequences in NIH ImageJ, and analyzed using the ImageJ plugin QuimP (version 18.02.01) (https://warwick.ac.uk/fac/sci/dcs/people/till_bretschneider/QuimP/) to count the number of cell membrane protrusions for each trophozoite. The parameters used for QuimP analysis are provided in Text S1. Only trophozoites that were completely visible in the field of view and did not touch other trophozoites for the duration of imaging were analyzed, which was typically 0 to 3 such trophozoites per field of view. Experiments were performed on three separate days to analyze a total of 40 trophozoites per EhCoactosin variant. The “motility map” output of QuimP was used to visually detect differences between the mutants, and statistical testing for differences was performed using the number of protrusions detected by QuimP for each trophozoite.

### Motility assay.

Trophozoites were treated with 5 μg/ml tetracycline for 24 h to induce overexpression of EhCoactosin variants and then resuspended in complete TYI medium. A pellet of 750 μl of log-phase Escherichia coli DH5α culture was mixed with the trophozoites, and 40 μl of this was then added to 80 μl of cold Matrigel (354234; Corning), which was allowed to solidify for 1 h at 37°C in 16-well dishes (112459; Grace BioLabs). After solidification, we took time-lapse transmitted light images of each mutant and control for 2 h with 2-min intervals using a 20× (0.5 NA) lens objective. Trophozoites moving under the Matrigel were consciously avoided. Each time sequence was acquired using an automated stage to capture images from 4 wells in a dish, each containing a separate parasite line, with 3 locations imaged per well. Images were exported in .tiff format and analyzed as image sequences using NIH ImageJ. Movement of individual trophozoites (on average, 7 per image sequence) was tracked with the ImageJ manual tracking plugin (https://ImageJ.nih.gov/ij/plugins/track/track.html). For hypothesis testing, the distance between the two farthest points was measured for each trophozoite. The average maximal distance moved by each parasite line on a given day was determined, and data from 6 independent experiments were combined.

### Phagocytosis assay.

Phagocytosis of healthy or apoptotic Jurkat T cells was assayed as previously described (69). Jurkat T lymphocytes were stained with 30 μM carboxyfluorescein succinimidyl ester (CFSE) at 37°C for 15 min. Residual CFSE was quenched by the addition of equal volumes of fetal bovine serum (FBS), and incubation for an additional 15 min. Cells were washed, resuspended in 7 ml RPMI growth medium, and exposed to UV light using a Fotodyne UV box for 15 min to induce apoptosis. Jurkat cells were then incubated for 2 h at 37°C in a 5.0% CO_2_ atmosphere. A suspension of 4.0 × 10^5^ labeled lymphocytes was then added to 2.0 × 10^5^ trophozoites in a final volume of 200 μl M199s, centrifuged, and incubated at 37°C for 20 min. Cells were washed twice in cold PBS with 20 mg/ml d-galactose, fixed in 300 μl 4% formaldehyde for 20 min, washed again, and resuspended in 500 μl PBS. Amoebic fluorescence was analyzed using a MACSQuant VYB flow cytometer. Phagocytic amoebae were defined as those with fluorescence levels above background (see Fig. S1), and data were expressed as a phagocytic index, defined as the percentage of phagocytic amoebae multiplied by the mean fluorescence of phagocytic amoebae. Phagocytosis of live Jurkat cells was examined as described above but without UV light exposure.

### Adherence assay.

Adherence to CHO cell monolayers was assayed as previously described with modifications (14). A 1.0-ml M199s suspension of trophozoites (2.0 × 10^5^/well) was distributed onto fixed CHO cell monolayers previously grown to confluence for 48 h on 24-well tissue culture plates. After incubation for 30 min at 37°C, nonadherent trophozoites were aspirated, and the cell monolayers were washed twice with 0.5 ml warm M199s. The wells were filled with 0.5 ml cold M199s with 20 mg/ml d-galactose, and the plate was placed on ice for 15 min to induce detachment of the remaining amoebae from the CHO monolayers. Nonadherent amoebae were then transferred to 1.5-ml tubes, resuspended in 100 μl M199s, and counted using a hemocytometer. Cell adherence was calculated as the percentage of adhered amoebae (EhCoactosin-overexpressing cells) relative to empty vector control cells.

### Multiple-sequence alignment.

A BLASTP search was performed using the EhCoactosin sequence as query against the NCBI nonredundant protein sequence database. Among the top 100 hits, sequences that aligned with the last 10 residues of EhCoactosin were downloaded in .FASTA format. The BLASTP search was then repeated against the “model organisms” database, and the identified homologues from human, mouse, zebrafish, and fruit fly were downloaded in .FASTA format. All the downloaded sequences were then aligned using Clustal Omega hosted at https://www.ebi.ac.uk/Tools/msa/clustalo/.

### Microscopy, statistical analysis, and figure preparation.

All imaging was performed using a Nikon Eclipse TE2000 microscope and objectives as specified. Images were captured with an EXiBlue fluorescence microscopy camera (QImaging, Canada) using NIS Elements Advanced Research (AR) software. Aside from the analyses performed with proteomics data (see Text S1), statistical analyses were performed using GraphPad Prism (version 8.0) and the statistical tests specified in each figure legend. Graphs were exported from GraphPad Prism as .eps files for incorporation into figures prepared using Adobe Illustrator.

### Data availability.

All proteomics and phosphoproteomics data generated in this work are provided as Data Set S1. The mass spectrometry proteomics data have also been deposited to the ProteomeXchange Consortium via the PRIDE partner repository with the data set identifier PXD018276.
