# Supplementary material for: Coactosin Phosphorylation Controls Entamoeba histolytica Cell Membrane Protrusions and Cell Motility
Source: mBio. 2020 Aug 4;11(4):e00660-20. doi: 10.1128/mBio.00660-20 (PMC7407079; doi:10.1128/mBio.00660-20)
Supplement: TABLE S1 [file mBio.00660-20-st001.pdf]

Supplemental Table 1. Proteins that are differentially expressed following *EhMSP-1* silencing.

Accession	Protein	Peptide	Peptide unique	EhMSP-1 (-) / WT	P value
EHI_182990A-p1	histone H2B, putative	2	2	0.83	9.8E-05
EHI8A_039580-t26_1-p1	hypothetical protein	2	2	0.85	0.0004
EHI_104560A-p1	cortexillin, putative	15	15	0.74	0.0005
EHI_183460A-p1	elongation factor 1 beta, putative	5	5	0.75	0.0006
EHI_020270A-p1	ubiquitin-activating enzyme, putative	8	8	0.70	0.0010
EHI_005890A-p1	60S ribosomal protein L3, putative	8	8	2.51	0.0013
EHI7A_159750-t26_1-p1	4Fe-4S binding domain containing protein	12	1	2.52	0.0014
EHI_153060A-p1	acyl-CoA synthetase, putative	2	2	0.68	0.0020
EHI_148480A-p1	WD domain containing protein	6	6	1.29	0.0020
EHI_068620A-p1	hypothetical protein	1	1	0.31	0.0024
EHI_076870A-p1	3-oxo-5-alpha-steroid 4-dehydrogenase domain-containing protein	6	6	0.73	0.0031
EHI_003010A-p1	ubiquitin-conjugating enzyme family protein	4	3	2.08	0.0037
EHI_169670A-p1	hypothetical protein	9	9	0.66	0.0040
EHI_132880A-p1	eukaryotic translation initiation factor 2 gamma subunit, putative	2	2	0.53	0.0042
EHI_092080A-p1	hypothetical protein, conserved	7	7	0.64	0.0047
EHI_176140A-p1	profilin, putative	5	5	1.34	0.0049
EHI_152650A-p1	type A flavoprotein, putative	30	30	1.47	0.0049
EHI_186840A-p1	actin-binding protein, cofilin/tropomyosin family	10	10	0.70	0.0053
EHI_035980A-p1	hypothetical protein	2	2	1.31	0.0054
EHI_186480A-p1	translation initiation factor eIF-5A, putative	12	12	1.16	0.0055
EHI_173410A-p1	mucin-2 precursor, putative	1	1	0.89	0.0057
EHI_148470A-p1	asparagine synthetase A, putative	8	8	1.29	0.0058
EHI_126140A-p1	60S ribosomal protein L9, putative	7	2	0.04	0.0059
EHI_117890A-p1	Rab family GTPase	1	1	0.52	0.0067
EHI_197010A-p1	hypothetical protein	8	8	0.65	0.0071
EHI_103270A-p1	T-complex protein 1 beta subunit, putative	8	8	1.62	0.0071
EHI_135470A-p1	adenylate kinase	11	11	1.55	0.0074
EHI_140720A-p1	myosin heavy chain	14	14	0.75	0.0083
EHI_155290A-p1	actinin-like protein, putative	3	3	1.50	0.0084
EHI_103260A-p1	NADPH-dependent FMN reductase domain containing protein	1	1	0.55	0.0085
EHI_104390A-p1	actin binding protein, putative	23	23	0.70	0.0087
EHI_068560A-p1	metallo-beta-lactamase family protein	2	2	0.70	0.0089
EHI_088070A-p1	ribosomal RNA methyltransferase, putative	4	4	1.27	0.0090
EHI_181140A-p1	hypothetical protein, conserved	2	2	0.63	0.0090
EHI_047810A-p1	hypothetical protein	28	4	1.26	0.0092
EHI_160940A-p1	aldehyde-alcohol dehydrogenase 2, putative	52	8	1.25	0.0092
EHI_048630A-p1	actin, putative	9	4	1.43	0.0095
EHI_186790A-p1	hypothetical protein, conserved	6	6	0.55	0.0097
