# Supplementary material for: Coactosin Phosphorylation Controls Entamoeba histolytica Cell Membrane Protrusions and Cell Motility
Source: mBio. 2020 Aug 4;11(4):e00660-20. doi: 10.1128/mBio.00660-20 (PMC7407079; doi:10.1128/mBio.00660-20)
Supplement: TABLE S2 [file mBio.00660-20-st002.pdf]

Supplemental Table 2: Phosphopeptides with a fold change either larger than 1.5 fold or smaller than 0.67 (CV < 30%) following *EhMSP-1* silencing.

Sequence	Master Protein Accessions	Protein	EhMSP-1 (-) / WT Bio Rep 1	EhMSP-1 (-) / WT Bio Rep 2	P value	CV (%)	Δ mass [ppm]	XCorr
[K].SALDNGTYSPLLSPTR.[R]	EHI_159500A [S244 (88.4) / S248 (88.4)]	Rho guanine nucleotide exchange factor	100	100	1E-17	0.0	-0.4	3.25
[K].AGGADYSFNTTSN.[-]	EHI_168340A [T146 (50) / S147 (50)]	actin-binding protein, cofilin/tropomyosin family - EhCoactosin	1.76	2.59	0.0172	27.0	0.28	3.62
*[K].KAGGADYSFNTTSN.[-]	EHI_168340A [S147 (99.9)]	actin-binding protein, cofilin/tropomyosin family - EhCoactosin	1.33	2.02	0.1163	29.1	-0.23	3.31
[K].ELDSDEEQKELER.[Q]	EHI_025370A [S410 (100)]	hypothetical protein, conserved	2.18	1.82	0.0305	12.5	-0.19	2.9
[R].KSPTIDEIK.[Q]	EHI_025430A [S134 (99.9)]	hypothetical protein	0.57	0.50	0.0593	9.5	0.23	2.5
[K].KTITPVVEDPCER.[I]	EHI_050150A [T662 (50) / T664 (50)]	HEAT repeat domain containing protein	0.45	0.63	0.0582	24.5	-0.69	3.9
[R].RRESVDYTNNK.[W]	EHI_110810A [S757 (100)]	unconventional myosin IB	0.50	0.50	0.0358	0.4	1.41	2.01

* [K].KAGGADYSFNTTSN.[-] (with 1 miscleavage), which was identified with the phosphorylation confidently located at S147, is included.
