# Supplementary material for: Coactosin Phosphorylation Controls Entamoeba histolytica Cell Membrane Protrusions and Cell Motility
Source: mBio. 2020 Aug 4;11(4):e00660-20. doi: 10.1128/mBio.00660-20 (PMC7407079; doi:10.1128/mBio.00660-20)

Supplemental Figure 1: Flow cytometry-based phagocytosis assay. Target cells (viable or UV-killed Jurkat lymphocytes) are fluorescently stained with CFSE, and incubated with *E. histolytica* trophozoites at 37°C. The samples are then analyzed by flow cytometry. Amoebas are distinguished from lymphocytes using forward scatter (FSC) and side scatter (SSC) characteristics, and phagocytic amoebae are defined as those with fluorescence greater than baseline. Data are then expressed as a phagocytic index, calculated as the mean fluorescence of phagocytic trophozoites x the percent of phagocytic trophozoites, and normalized to the empty vector control to enable combined analysis of experiments performed on different days. The figure shows example data. (A) SSC vs. FSC for control sample with *E. histolytica* trophozoites alone. (B) SSC vs. FSC for a sample with viable CFSE-labeled Jurkat lymphocytes alone. The gate shown in (A) and (B) identifies the majority of trophozoites while excluding non-phagocytosed host cells. (C) Example fluorescence histograms for amoebas identified by the gate in (A) and (B) for amoebas incubated alone and for each cell line incubated with CFSE-labeled Jurkat cells, shown either as an overlay on the left or as aligned histograms on the right. The M1 gate identifies non-phagocytic trophozoites, and the M2 gate identifies phagocytic trophozoites. Red = amoeba baseline fluorescence (i.e. no labeled lymphocytes); Blue = Empty Vector control; orange = Coac-WT; light green = Coac-A; Dark green = Coac-D.

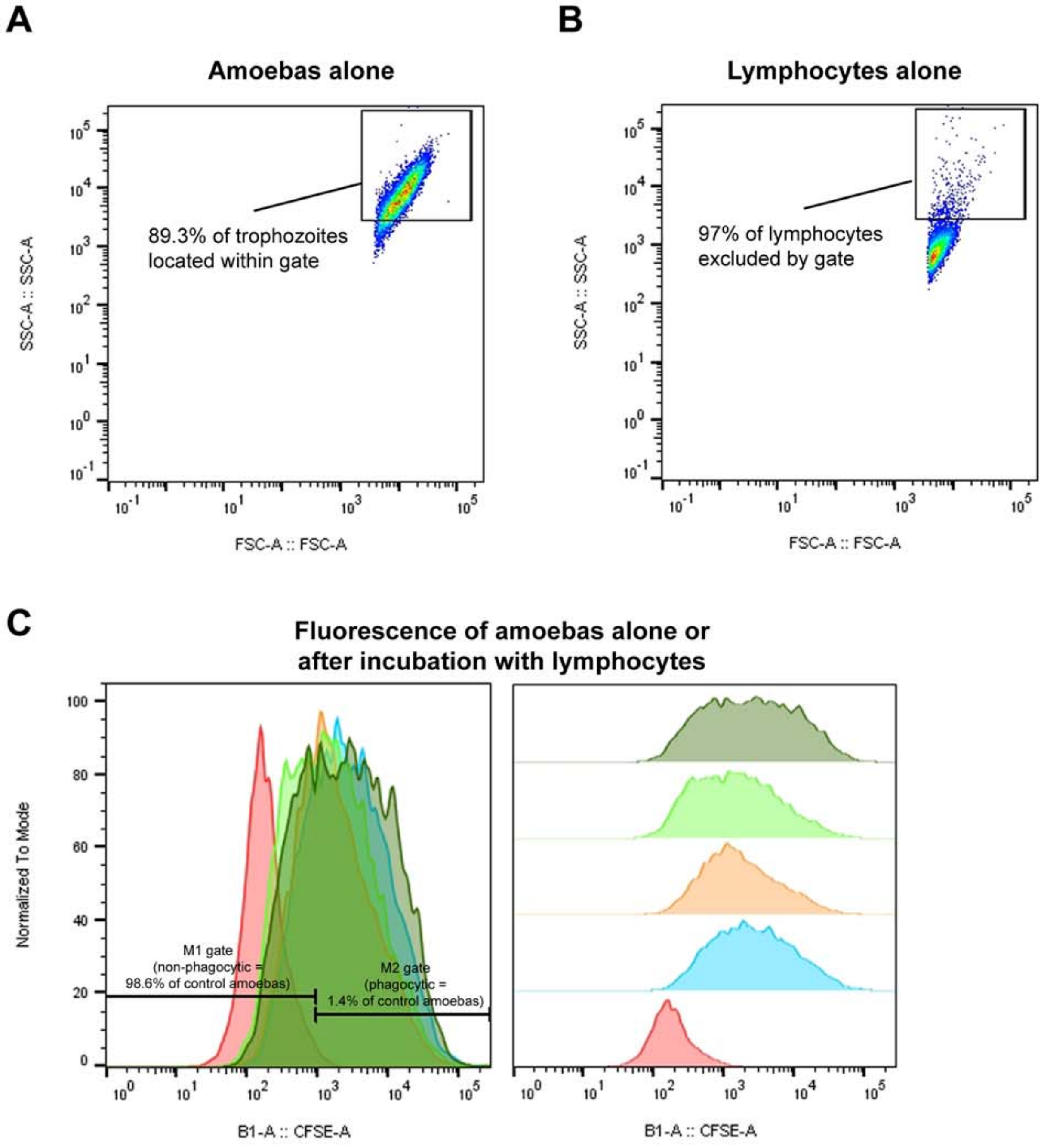

Supplement: FIG S1 [file mBio.00660-20-sf001.pdf]
