# Supplementary material for: Coactosin Phosphorylation Controls Entamoeba histolytica Cell Membrane Protrusions and Cell Motility
Source: mBio. 2020 Aug 4;11(4):e00660-20. doi: 10.1128/mBio.00660-20 (PMC7407079; doi:10.1128/mBio.00660-20)

Supplemental Figure 2: Multiple sequence alignment of Coactosin homologs from selected species. The species names are indicated on the left. The *E. histolytica* Coactosin row is marked with a red border and the amino acid position corresponding to S147 of EhCoactosin is marked with a black box and an asterisk.

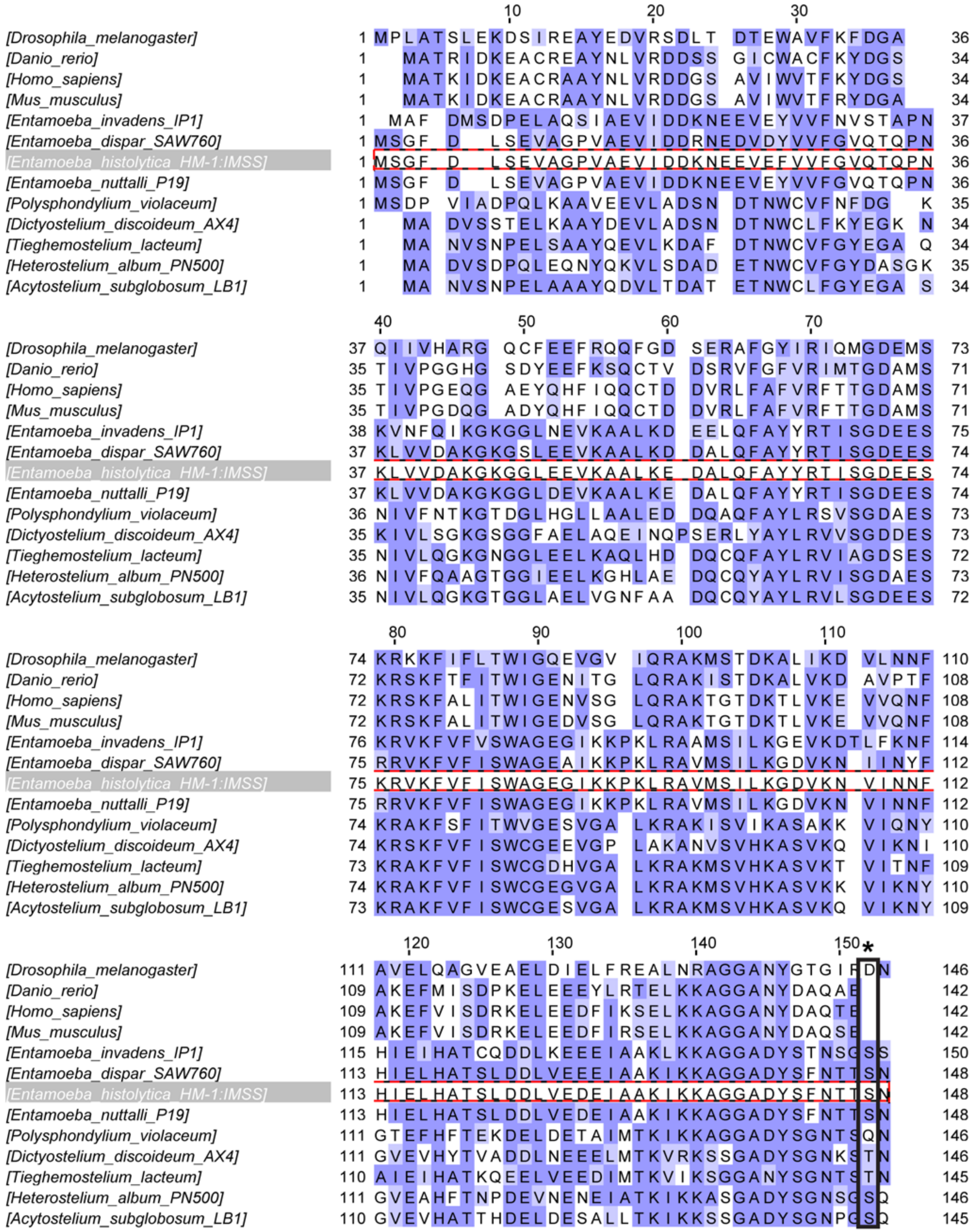

Supplement: FIG S2 [file mBio.00660-20-sf002.pdf]
