# Supplementary material for: Coactosin Phosphorylation Controls Entamoeba histolytica Cell Membrane Protrusions and Cell Motility
Source: mBio. 2020 Aug 4;11(4):e00660-20. doi: 10.1128/mBio.00660-20 (PMC7407079; doi:10.1128/mBio.00660-20)
Supplement: TEXT S1 [file mBio.00660-20-s0001.pdf]

Supplemental Methods.

Quantitative and phosphoproteomics detailed methods.

Trypsin digestion.

The *E. histolytica* cell pellets (*EhMSP-1* silenced (EhMSP-1 (-)) and WT) were reconstituted in 0.5 ml of 8 M urea with a minimum of 2 X phosphatase inhibitor (PI) (Halt™ Phosphatase Inhibitor Single Use Cocktail (100 X))). The suspension was sonicated on ice with three bursts for 20 seconds each at 1-min intervals. Protein concentration was determined by bicinchoninic acid assay. The solution containing a minimum of 20 mg of protein was diluted in 20 mM HEPES, pH 8/PI to 100 ml (final PI concentration of a minimum of 2 ×). Reduction of disulfides was achieved by adding dithiothreitol to the protein extract to a final concentration of 4.5 mM and incubating at 55 ^{o}C for 30 min. Alkylation was then carried out in the dark at a final concentration of 10 mM iodoacetamide at room temperature for 15 min. The solution was diluted 4-fold with 20 mM HEPES pH 8.0//PI (final PI concentration of a minimum of 2 ×) followed by trypsin digestion (Promega Sequence Grade Modified Trypsin V511) with an enzyme to substrate ratio of 1:50. Digestion was carried out overnight and was then stopped by adding trifluoroacetic acid (TFA) to a final concentration of 1% (the pH was lowered to below 3). The tryptic peptides were purified using Sep-Pak C18 cartridges (Waters, Milford, MA), and then lyophilized.

Stable isotope dimethyl-labeling.

The lyophilized peptides from EhMSP-1 (-) and WT samples were reconstituted in 1.88 ml 1M HEPES pH 7.5 and subjected to dimethyl-labeling as previously described (1-3). Briefly, 20 µl of 10% formaldehyde (diluted from a 37% solution; Sigma) and 40 µl of 500 mM sodium cyanoborohydride ($NaCNBH_3$) (diluted from a 5 M solution in 1 M NaOH; Sigma) were added to the WT peptides, whereas 20 µl of 10% d2-formaldehyde (diluted from a 20% solution, 98% D2; Cambridge Isotope Laboratories, MA) and 40 µl of 500 mM sodium cyanoborodeuteride $NaCNBD_3$ in 1 M NaOH (98.7% D2; CDN Isotopes, Canada) were added to the EhMSP-1 (-) peptides. The peptide mixtures were incubated at room temperature for 1 h. The labeling and incubation steps were repeated once, and the digests were tested by short mass spectrometry runs and database searches to ensure that > 99% of the peptides were labeled. The peptides from EhMSP-1 (-) and WT were acidified by TFA to a final concentration of 7% (pH < 3), combined, cleaned by Sep-Pak tC18 column, an aliquot from each sample was saved for subsequent SCX fractionation for total protein expression profiling, and the reminder of the solution was lyophilized for later phosphopeptide enrichment.

Strong cation exchange chromatography.

2.5 mg of WT and EhMSP-1 (-) labeled peptides were combined and resuspended in 750 µl buffer A (10 mM potassium phosphate (pH 2.8) and 25% acetonitrile (CH3CN)). An aliquot of 50 µl was fractionated on a PolySULFOETHYL A (silica SCX) cation exchange TopTip (0-200 µL sized, PolyLC, MD) by step elution (100 µL) with salt-cuts of 0, 20, 25, 30, 35, 40, 45, 50, 55, 60, 65, 70, 75, 80, 85, 90, 95, 100, 200, and 350 mM of KCl in buffer A. 15 ul of the eluates was dried down, ziptipped (Pierce), and dried again. The dried peptides were kept at -80°C until liquid chromatography/mass spectrometry (LC/MS) analysis.

Phosphopeptide enrichment.

Purified labeled peptides were reconstituted in 0.1% TFA 50% acetonitrile (CH_3CN) to a concentration of 1 mg/ml and then incubated for 1 h on an end-over-end mixer with immobilized metal affinity chromatography (IMAC) bead slurry (Sigma; PHOS-Select TM Iron Affinity Gel; 10 mg of peptides /ml 50% slurry) that were pre-washed with 0.1% TFA 50% CH_3CN thrice. The beads were washed with 10× bead volume of 0.1% TFA 50% CH_3CN twice and then with water once. The phosphopeptides were eluted with 10× bead volume of 1% NH_4OH (pH 11) at room temperature for 5 min twice. The eluate was lyophilized. The unbound fraction of peptide solution was adjusted to a final concentration of 50% MeCN/25% lactic acid and 0.1% TFA, and subjected to further phosphopeptide enrichment using TiO_2 beads (GL Sciences, Tokyo), which were conditioned with 2 ml of 80% MeCN/0.5% TFA (A) and washed with B (20% lactic acid (2.0 M) in A) for equilibration. The peptides were allowed to bind to the beads for 1 h. The beads were washed with B, then with A twice, followed by an incubation with 400 µl 5% NH4OH (pH 11) for 10 min. The eluate was collected and the beads were further eluted with 400 µl 5% pyrrolidine aqueous solution (4, 5). The eluates from IMAC and TiO2 enrichment were dried down and purified by STAGE tips or Ziptips.

Liquid chromatography-tandem mass spectrometry (LC-MS/MS).

The dried peptides from phosphopeptide enrichment and each of the 20 SCX fractions were reconstituted with 2.5% CH_3CN/2.5% formic acid (FA) and analyzed by nano-scale LC/MS on an LTQ-Orbitrap Discovery coupled to a Surveyor MS Pump Plus (Thermo Fisher Scientific, MA). Digests were loaded directly onto a 100 µm x 120 mm capillary column, which was laser pulled ~3 µm orifice and packed with MAGIC C18 (5 µm particle size, 20 nm pore size, Michrom Bioresources, CA), at a flow rate of 500 nl/min, and peptides were separated by a gradient of 2.5 – 5% CH_3CN /0.1% FA in 5 min, 5-35% CH_3CN /0.1% FA in 100 min, 35-100% CH_3CN/0.1% FA in 5 min, 100%/0.1% FA CH_3CN in 10 min followed by an immediate return to 2.5% CH_3CN/0.1% FA and a hold at 2.5% CH_3CN/0.1% FA until the next injection. Peptides were introduced into the linear ion trap via a nanospray ionization source with a spray voltage of 1.8 kV. Mass spectrometry data were acquired in a data-dependent "Top 5" acquisition mode with lock mass function activated (protonated polydimethylcyclosiloxane $(Si(CH_3)_2O))_6$; *m/z* 371.1012)), in which a Orbitrap survey scan from *m/z* 360-1600 at 30,000 (FWHM) resolution was paralleled by 5 collision-induced dissociation (CID) tandem mass spectrometry (MS/MS) scans of the most abundant ions in the LTQ. MS/MS scans were acquired with the following parameters: isolation width: 2 *m/z*, normalized collision energy: 35%, Activation Q: 0.250 and activation time = 30 ms. Review mode for FTMS master scans was enabled. Dynamic exclusion was enabled (repeat count: 2; repeat duration: 30 sec; exclusion list size: 180; exclusion duration: 60 sec). The minimum threshold was 500. Singly charged ions were excluded for MS/MS. Singly charged and unassigned charge state ions were excluded for MS/MS. Charge state screening, monoisotopic precursor selection, and preview mode for FTMS master scans were enabled.

Peptide identification.

Product ion spectra were searched using the SEQUEST search engines implemented in Proteome Discoverer 2.2 (Thermo Fisher Scientific, Waltham, MA, USA) in the Processing workflow against curated protein databases (6) (AmoebaDB-

34_EhistolyticaHM1IMSS_AnnotatedProteins.fasta, AmoebaDB-34_EhistolyticaHM1IMSS-A_AnnotatedProteins.fasta, AmoebaDB-34_EhistolyticaHM1IMSS-B_AnnotatedProteins.fasta; downloaded in November, 2017). The 20 raw files generated from SCX fractions of the total protein expression profiling experiment were processed as one input file, and the IMAC and TiO_2 separate runs were also combined as one search input. Search parameters were as follows: (1) full trypsin enzymatic activity; (2) maximum missed cleavages = 2; (3) mass tolerance at 20 ppm and 0.8 Da for precursor ions and fragment ions, respectively; (4) dynamic modifications on methionine (+15.995 Da: oxidation) and on serine/threonine/tyrosine (+79.966 Da: phosphorylation); and (5) static modification on cysteine (+57.022 Da: carbamidomethylation) and static isomeric dimethyl modifications on lysine and N-terminus (+28.031 Da C(2) H(4): for WT and +34.069 Da C(2)D(6)H(-2) for EhMSP-1 (-)). ptmRS node was used to assess the localization of phosphorylation sites. Percolator was included in the workflow to limit the false positive rates to less than 1% in the data set**.** The search result files were imported into the Scaffold 4.3 (Proteome Software, Portland, OR) for sequence annotation.

Quantification and statistical analysis of proteomics data.

Three independent biological replicates, BioRep 1, 2 and 3, were analyzed for the *EhMSP-1* silenced differential proteome experiments whereas for the phosphopeptide analysis, two biological replicates, Bio Rep 1 and Bio Rep 2, were subjected to the phosphopeptide enrichment workflow. An additional replicate was derived from Bio Rep 2 (starting from peptide digestion, labeling, and peptide enrichment to mass spectrometry analysis), which was treated as a technical replicate for Bio Rep 2 in the Proteome Discoverer analysis workflow. The relative abundances of peptides EhMSP-1 (-)/WT were quantified by the Precursor Ion Quantifier node. In the Consensus workflow, parameters were set as follows: (1) both unique and razor peptides were used for quantification; (2) Reject Quan Results with Missing Channels: False; (3) Normalization mode: "Total Peptide Amount" for the differential proteome analysis and "None" for the phosphopeptide analysis; (4) Precursor Abundance Based on: Area; and (5) Scaling Mode was set on "All Average". Nested design was used for the analysis of biological replicates (Respective biological replicates were set in the Study Factor). Ratio calculation was Summed Abundance Based for the differential proteome analysis (normalized for total peptide amount) and "Pairwise Ratio Based" for phosphopeptide analysis. For Hypothesis testing, ANOVA (individual protein) was used for the differential proteome analysis and "Background Based" t-test was used for phosphopeptide analysis (not normalized to total peptide). The individual protein ANOVA evaluated the normalized intensities across all replicates whereas "Background Based" evaluated the ratios. p-values (t-test) and adjusted p-values (Benjamini-Hochberg method) were calculated accordingly. Only proteins identified in all biological replicates in respective experiments were kept. All the protein identification and quantification information (<1% FP; with protein grouping enabled) was exported from the Proteome Discoverer result files to Excel spreadsheets (**Supplemental** Tables S1 and S2). Ratios were then imported into the JMP Pro 13 (SAS Institute, Cary, NC) to construct heat maps, and fold change ($\log_2$) and p-values ($-\log_{10}$) into GraphPad Prism 8 (GraphPad Software Inc., CA) for constructing volcano plots. The raw intensity values were imported from XCalibur into GraphPad Prism 8 after extracting the monoisotopic ion $\pm$ 0.01 *m/z* window of the target ion and averaging scans across the peak of elution.

Method used for protrusion tracking with QuimP.

1. Open image from Quimp plugin unchecking the “new format” option
2. Each cell outline was detected using the following BOA options:
3. Node spacing: 2
4. Final Shrinkage: 6
5. Sample tan: 5
6. Sample norm: 18
7. ECM: Ran as default. Utilized individual .PaQP files
8. ANA: Ran with 2uM cortex. Utilized individual .PaQP files
9. Q analysis: ran with 1200 resolution.
   (All the other options in Quimp were kept as default).
10. Open motility_map.maqp in imagej using “import text image”
11. Perform Gaussian Blur with sigma 2.
12. Save as “text image”
13. Edit format of the text image so that it is readable by Quimp again with a text editor. Mainly, converting “tab-separated” file into “comma-separated” file and removing the last line break.
14. Run Qconf to .paqp
15. Run Protrusion tracking with Drop 0.8 and Sen 0.5.
16. Open the “Protstat” output in MS Excel
17. Sort by “frame number”. As the X-axis of motility maps denotes node position along cell outline, same protrusion can be detected twice if they originate at the earliest or the latest nodes. Sorting by “frame number” followed by inspecting “position” allows detection of such protrusions. They can be omitted from counting.

Count the number of protrusions of each trophozoite.

References:

1. Hsu JL, Huang SY, Chow NH, Chen SH. 2003. Stable-isotope dimethyl labeling for quantitative proteomics. Anal Chem 75:6843-6852.
2. Boersema PJ, Raijmakers R, Lemeer S, Mohammed S, Heck AJ. 2009. Multiplex peptide stable isotope dimethyl labeling for quantitative proteomics. Nat Protoc 4:484-494.
3. Smith KP, Fields JG, Voogt RD, Deng B, Lam YW, Mintz KP. 2015. Alteration in abundance of specific membrane proteins of *Aggregatibacter actinomycetemcomitans* is attributed to deletion of the inner membrane protein MorC. Proteomics 15:1859-1867.
4. Wojcechowskyj JA, Lee JY, Seeholzer SH, Doms RW. 2011. Quantitative phosphoproteomics of CXCL12 (SDF-1) signaling. PLoS One 6:e24918.
5. Saleem RA, Rogers RS, Ratushny AV, Dilworth DJ, Shannon PT, Shteynberg D, Wan Y, Moritz RL, Nesvizhskii AI, Rachubinski RA, Aitchison JD. 2010. Integrated phosphoproteomics analysis of a signaling network governing nutrient response and peroxisome induction. Mol Cell Proteomics 9:2076-2088.

6. Aurrecoechea C, Barreto A, Brestelli J, Brunk BP, Caler EV, Fischer S, Gajria B, Gao X, Gingle A, Grant G, Harb OS, Heiges M, Iodice J, Kissinger JC, Kraemer ET, Li W, Nayak V, Pennington C, Pinney DF, Pitts B, Roos DS, Srinivasamoorthy G, Stoeckert CJ, Jr., Treatman C, Wang H. 2011. AmoebaDB and MicrosporidiaDB: functional genomic resources for Amoebozoa and Microsporidia species. Nucleic Acids Res 39:D612-619.
